# Neuroquantification enhances the radiological evaluation of term neonatal hypoxic-ischaemic cerebral injuries

**DOI:** 10.4102/sajr.v27i1.2728

**Published:** 2023-12-26

**Authors:** Shalendra K. Misser, Nobuhle Mchunu, Jan W. Lotz, Lisa Kjonigsen, Aziz Ulug, Moherndran Archary

**Affiliations:** 1 Department of Radiology, Lake Smit and Partners Inc., Durban, South Africa; 2 Department of Radiology, Faculty of Health Sciences, University of KwaZulu-Natal, Duban, South Africa; 3 Biostatistics Research Unit, South African Medical Research Council, Durban, South Africa; 4 Department of Statistics, Faculty of Science, School of Mathematics, Statistics and Computer Sciences, University of KwaZulu-Natal, Pietermaritzburg, South Africa; 5 Department of Radiodiagnosis, Faculty of Sciences, Stellenbosch University, Cape Town, South Africa; 6 Medtech, Oslo, Sweden; 7 Cortechs Labs, San Diego, United States of America; 8 Department of Pediatrics, Faculty of Health Sciences, University of KwaZulu-Natal, Durban, South Africa

**Keywords:** neuroquantification, hypoxic-ischaemic, cerebral, term neonatal, magnetic resonance, artificial intelligence, brain volumetry

## Abstract

**Background:**

Injury patterns in hypoxic-ischaemic brain injury (HIBI) are well recognised but there are few studies evaluating cerebral injury using neuroquantification models.

**Objectives:**

Quantification of brain volumes in a group of patients with clinically determined cerebral palsy.

**Method:**

In this retrospective study, 297 children with cerebral palsy were imaged for suspected HIBI with analysis of various cerebral substrates. Of these, 96 children over the age of 3 years with a clinical diagnosis of cerebral palsy and abnormal MRI findings underwent volumetric analyses using the NeuroQuant^®^ software solution. The spectrum of volumetric changes and the differences between the various subtypes (and individual subgroups) of HIBI were compared.

**Results:**

Compared with the available normative NeuroQuant^®^ database, the average intracranial volume was reduced to the 1st percentile in all patient groups (*p* < 0.001). Statistically significant differences were observed among the types and subgroups of HIBI. Further substrate volume reductions were identified and described involving the thalami, brainstem, hippocampi, putamina and amygdala. The combined volumes of five regions of interest (frontal pole, putamen, hippocampus, brainstem and paracentral lobule) were consistently reduced in the Rolandic basal ganglia-thalamus (RBGT) subtype.

**Conclusion:**

This study determined a quantifiable reduction of intracranial volume in all subtypes of HIBI and predictable selective cerebral substrate volume reduction in subtypes and subgroups. In the RBGT subtype, a key combination of five substrate injuries was consistently noted, and thalamic, occipital lobe and brainstem volume reduction was also significant when compared to the watershed subtype.

**Contribution:**

This study demonstrates the value of integrating an artificial intelligence programme into the radiologists’ armamentarium serving to quantify brain injuries more accurately in HIBI. Going forward this will be an inevitable evolution of daily radiology practice in many fields of medicine, and it would be beneficial for radiologists to embrace these technological innovations.

## Background

Hypoxic-ischaemic brain injury (HIBI) is an important cause of cerebral palsy^1^ worldwide, across the divide between high- and low- to-middle-income countries (LMICs).^2^ The patterns of injury ascribed to HIBI are well recognised,^3,4,5,6,7^ and the pathophysiology of these has been extensively studied through animal studies.^8,9,10,11,12^ Some of the injuries are related to the early phase of cell damage encountered immediately after a sentinel event or around the perinatal period. Further apoptotic injuries result from glutamate-induced delayed and secondary injuries that can be ongoing for days to weeks after the insult. These may occur with the remodelling processes innate to the developing brain.^13^ The result of perinatal HIBI is the structural damage of substrates that may be directly injured and others that are secondarily injured in these processes.^14^

Injuries sustained in the perinatal period are reflected in specific cerebral regional destruction that persists throughout the child’s life. Assessment of normal and abnormal cerebral development and myelination is possible with standard MRI.^15,16^ The traditionally reported HIBI patterns seen on MRI pertain to the cerebral watershed territories, deep nuclear structures, periventricular white matter and multilobar cystic encephalopathy.^14^ In order to improve the reliability of MRI in the setting of HIBI, one must ensure the adequate preparation of patients before scanning, due attention to the use of correct imaging protocols and the recruitment of homogeneous cohorts.^17^ Neuroquantification software and artificial intelligence (AI) algorithms have been developed for MRI to obtain a more accurate depiction of the total burden of injury in individual patients.^15^

There have been few studies evaluating cerebral injury using volumetric quantification models. One such algorithm has been developed specifically for volumetry in cerebral palsy patients.^18^ Such AI programmes aim to improve diagnosis, stratification of brain injuries and correlation with functional outcome. This study aimed to quantify the various cerebral substrate injuries and brain volumes in a group of patients with clinically determined cerebral palsy, using accredited neuroquantification software. Specific areas of interest corresponding to those described in HIBI were targeted for volumetric measurement.

## Materials and methods

A composite database was accumulated over a 6-year period, comprising 297 children with clinically recorded term neonatal hypoxic-ischaemic encephalopathy (HIE) and a clinical diagnosis of cerebral palsy, imaged by MRI in the chronic phase of evolution. Multiple sequences were performed on 1.5 T Siemens Scanners, including sagittal volumetric T1-weighted, coronal and axial T2-weighted, axial fluid attenuated inversion recovery (FLAIR), susceptibility weighted imaging (SWI), diffusion weighted imaging (DWI) and apparent diffusion coefficient (ADC), and inversion recovery sequences. All MRI studies were retrospectively and independently reviewed by the principal investigator (S.K.M.) and co-investigator (J.L.). Using the classification as proposed by Misser et al.,^14^ each study was evaluated to identify the HIBI subtype and record the degree of involvement of the thalami, hippocampi, basal ganglia, white matter, cortical regions, brainstem and several other critical substrates. Each investigator individually graded the selected substrates. Interobserver discordances were reviewed, discussed and resolved by the two primary readers. Categorisation of the four HIBI subtypes was undertaken, and correlation with the degree of substrate injury for each subtype and their various subgroups of injury were tabulated.

**FIGURE 1:**
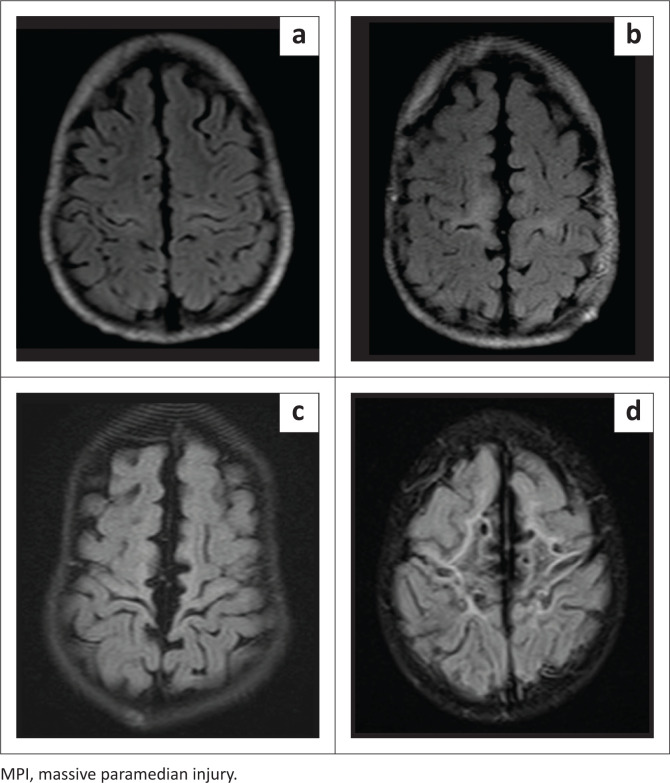
Axial FLAIR images in patients with the Rolandic basal ganglia-thalamus subtype demonstrating the classification^19^ of the four subgroups of paramedian extension of perirolandic injury, (a) Mild, (b) Moderate, (c) Severe, (d) MPI.

**FIGURE 2:**
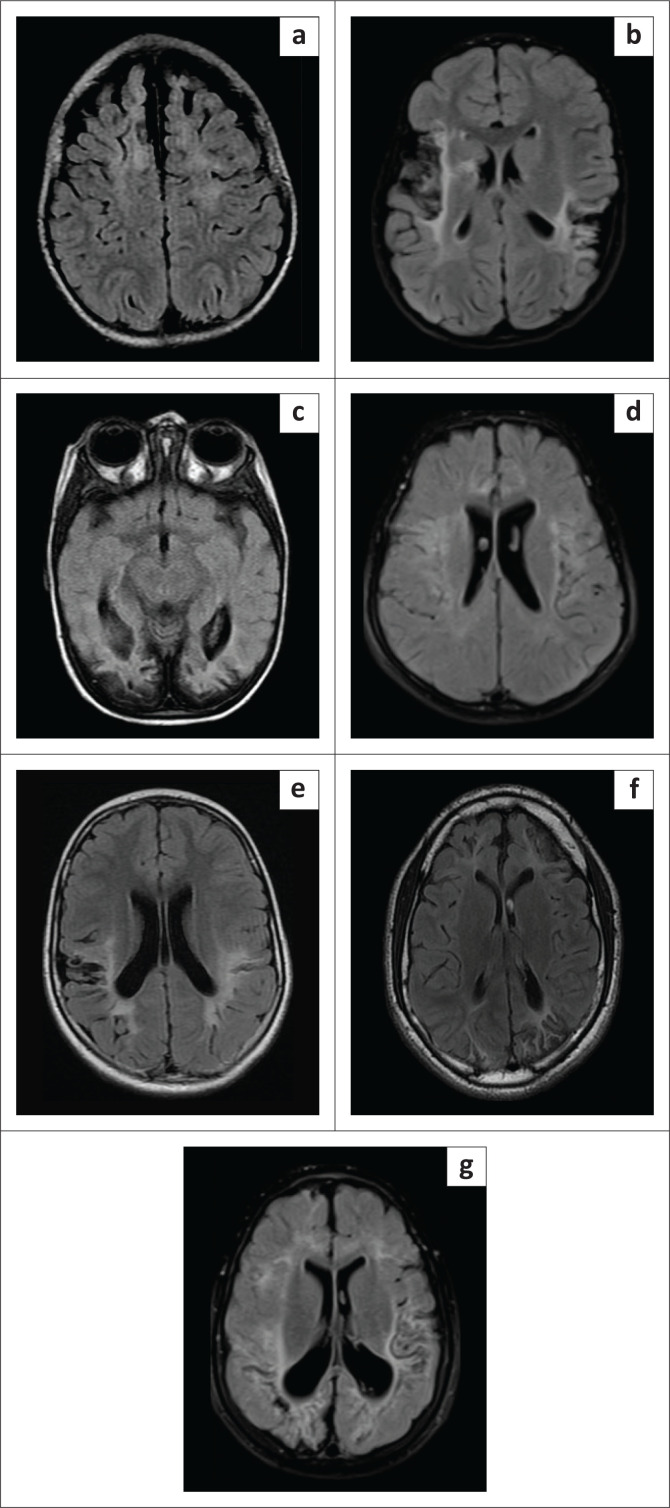
Axial FLAIR images in patients with parasagittal or partial prolonged subtype showing the classification^21^ of the seven subgroups as per the watershed zones involved, (a) Anterior, (b) Perisylvian, (c) Posterior, (d) Anterior + Perisylvian, (e) Perisylvian + posterior, (f) Anterior + posterior, (g) All three zones.

The four major HIBI subtype classifications in this study:

- The central or Rolandic basal ganglia-thalamus (RBGT) subtype, which includes patients with isolated high metabolic area injury (sensorimotor cortex, ventral posterior lateral (VPL) nucleus of the thalamus, posterior putamen and hippocampus), is subdivided into four gradations of injury, based on the degree of destruction of the parasagittal cortex^19^ (Figure 1). These include the mild, moderate, severe and massive paramedian injury (MPI) subgroups.
- The parasagittal or partial prolonged pattern of injury related to the destruction of the cortex and subcortical white matter in the named watershed areas with seven subgroups and/or combinations of these areas^20,21^ (Figure 2).
- The mixed pattern of injury incorporates a combination of high metabolic zone and watershed territory involvement.^14^
- Multicystic encephalomalacia follows global brain injury with spongiosis of multiple lobes and cystic change.^14^

### Parasagittal scoring

The parasagittal scores were calculated for each patient with substrate injuries at the parasagittal structures measured according to a previously reported grading system.^19^ These included the involvement of the paracentral lobule (PCL), supplementary motor areas, superior frontal gyrus, precentral gyrus, postcentral gyrus and the degree of interhemispheric fissure widening. These five specific parasagittal features were each scored from 0 to 3 using a simple scoring system: 0 = no injury, 1 = mild injury (less than a third of the structure was injured), 2 = moderate injury (more than one-third, but less than two-thirds of the structure was injured) and 3 = severe injury (more than two-thirds, with up to near-complete destruction of the substrate). The sum of these five measured values added to a potential maximum parasagittal score of 15.

### Quantitative volumetric analysis

**FIGURE 3:**
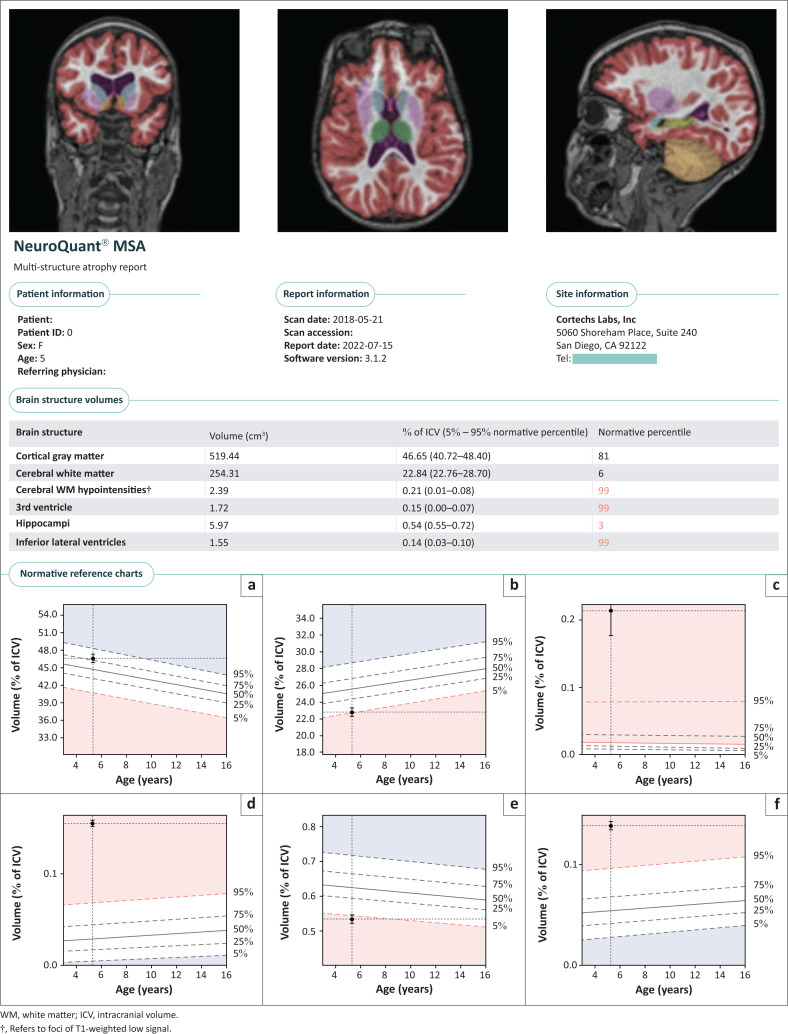
Algorithm used for volumetric analysis of brain structures in three planes and sample patient output of NeuroQuant® (Cortechs Labs, Inc., San Diego, California, United States) with detailed substrate evaluation and percentile grading, (a) cortical gray matter, (b) cerebral white matter, (c) cerebral WM hypointensities, (d) 3rd ventricle, (e) hippocampi, (f) inferior lateral ventricles.

The substructure volumes were measured using the software solution NeuroQuant^®^ (Cortechs Labs, Inc., San Diego, CA, United States [US]). This AI tool is the first FDA- approved software for volumetric MRI processing. NeuroQuant^®^ calculates the volume of different brain substructures and compares those to a large normative age- and gender-matched database to determine whether the degree of brain volume loss is statistically significant for patient age. Each patient is then allocated a percentile ranking for every volumetric measurement, compared to age-matched controls. A sample of the volumetric data acquisition process is displayed in Figure 3. These measurements were independently overseen by co-investigators (L.K. and A.U.), who were blinded to any clinical details or radiological diagnoses. Because comparable age-matched atlases are not available for children under the age of 3 years, they were therefore excluded from the volumetric analyses.

### Statistical analysis

The Kolmogorov-Smirnov test was used to assess normality assumptions of the continuous variables. All variables violated the normality assumptions and were thus expressed as medians with interquartile ranges (IQR). The Kruskal-Wallis test, a non-parametric version of the ANOVA (analysis of variance) test, was used to analyse differences between the medians of two or more groups. The Mann-Whitney U test was used to analyse differences between the two groups of HIBI subtype 2 (watershed pattern). A two-tailed *p* < 0.05 indicated statistical significance. All statistical analyses were conducted using SAS, version 9.4 (SAS Institute). Box plot graphs were generated using GraphPad Prism Software 8.0.2 (GraphPad Software, La Jolla, CA, US).

### Ethical considerations

All procedures performed in studies involving human participants were in accordance with the ethical standards of the institutional and/or national research committee and with the 1964 Helsinki Declaration and its later amendments or comparable ethical standards. This retrospective study was approved by the Biomedical Research Ethics Committee of the University of KwaZulu-Natal (UKZN) (BREC/00001036/2020).

## Results

A total of 96 cases were recruited for the study. Five cases were excluded as the imaging sequences did not process without errors on the neuroquantification software programme. The youngest child imaged was 3 years (owing to the threshold for the volumetric evaluation), and the oldest was 9 years of age (median age = 5 years). Categorisation of the four HIBI subtypes was undertaken, and correlation with the degree of substrate injury for each subtype and their various subgroups of injury were tabulated. There was a high degree of interobserver agreement between the two readers, with a weighted kappa of > 0.8.

There were three predominant patterns of HIBI, as displayed in Table 1. The fourth subtype was multicystic encephalomalacia, only seen in one patient. Thirty-four demonstrated the RBGT/central pattern of injury, and these were further subdivided into mild (*n* = 11), moderate (*n* = 9), severe (*n* = 4) and MPI (*n* = 10). Watershed-only injuries were identified in 35 individuals, and they were classified into seven subgroups, with anterior (*n* = 2), perisylvian (*n* = 1), posterior (*n* = 1), anterior + perisylvian (*n* = 3), perisylvian + posterior (*n* = 3), anterior + posterior (*n* = 3) and all three zones involved (*n* = 22). Twenty-three patients demonstrated a mixed watershed/RBGT pattern.

**TABLE 1:** Volumetric distribution by hypoxic-ischaemic brain injury patterns.

Variable	RBGT (*n* = 4)		Partial prolonged (*n* = 35)		Mixed pattern (*n* = 26)		Total† (*n* = 96)		*p*
	Median	IQR	Median	IQR	Median	IQR	Median	IQR	
Age (years)	6	4–9	5	4–9	5	3–6	5	4–7	0.089
Parasagittal Score	3	2–5	10	7–13	7	4–11	6	3–11	< 0.001
Amygdala Vol	2	2–3	3	2–3	2	2–3	2	2–3	0.310
Amygdala %	1	1–5	33	6–80	3	1–22	6	1–33	< 0.001
Basal Ganglia Vol	21	15–24	20	15–25	20	15–23	21	15–24	0.907
Basal Ganglia %	27	1–86	88	9–99	39	1–99	58	1–99	0.050
Brainstem Vol	14	11–17	14	13–16	14	11–15	14	11–16	0.384
Brainstem %	15	2–85	99	75–99	31	7–77	58	7–99	< 0.001
Caudate Vol	8	5–9	7	5–10	7	6–8	7	5–9	0.612
Caudate %	88	47–99	99	96–99	99	40–99	99	67–99	0.039
Forebrain Vol	843	652–971	597	465–672	592	525–923	658	525–899	< 0.001
Forebrain %	21	1–60	1	1–5	1	1–30	1	1–31	0.009
Frontal Vol	4	2–5	2	1–3	2	1–4	2	1–4	0.002
Frontal %	20	4–49	2	1–9	4	1–38	4	1–37	0.008
Cortical Vol	513	367–595	-	-	292	214–595	364	213–545	< 0.001
Cortical %	59	4–93	1	1–5	1	1–73	3	1–79	0.001
Hippocampus Vol	6	5–7	6	5–7	6	4–6	6	5–7	0.227
Hippocampus %	3	1–20	67	3–99	2	1–76	8	1–84	0.001
Intracranial Vol	1167	951–1331	909	777–974	900	827–1273	962	838–1229	0.001
Intracranial %	1	1–8	1	1–1	1	1–2	1	1–1	< 0.001
Paracentral Vol	9	6–12	4	3–7	6	4–9	7	4–11	0.001
Paracentral %	21	1–73	1	1–2	1	1–26	1	1–64	0.003
Parietal Vol	127	99–147	67	47–93	81	53–146	93	53–136	< 0.001
Parietal %	71	9–97	1	1–18	9	1–91	14	1–91	0.001
Primary Motor Vol	32	26–37	17	12–23	20	14–37	23	15–33	< 0.001
Primary Motor %	97	66–99	4	1–74	64	1–97	65	1–98	< 0.001
Primary Sensory Vol	28	21–31	17	11–22	19	12–29	21	12–29	< 0.001
Primary Sensory %	97	78–99	26	1–98	79	1–99	82	1–99	0.014
Putamen Vol	10	7–13	9	5–12	8	7–12	10	6–12	0.587
Putamen %	1	1–50	53	1–95	1	1–59	1	1–68	0.096
Occipital Vol	51	35–60	28	19–33	27	21–62	32	23–58	0.001
Occipital %	10	1–46	1	1–1	1	1–34	1	1–26	0.005
Thalamus Vol	11	9–13	12	9–14	12	10–14	12	10–15	0.364
Thalamus %	1	1–11	77	8–99	66	1–99	32	1–99	< 0.001
Ventricle Vol	36	23–57	35	22–60	34	20–67	35	21–59	0.459
Ventricle %	99	94–99	99	99–99	99	99–99	99	98–99	0.420

Note: % = Percentile ranking; vol = volume measured in cm^3^.

RBGT, Rolandic Basal Ganglia-Thalamus; IQR, interquartile range

† , Total includes one patient with multicystic encephalomalacia as per the earlier results.

**FIGURE 4:**
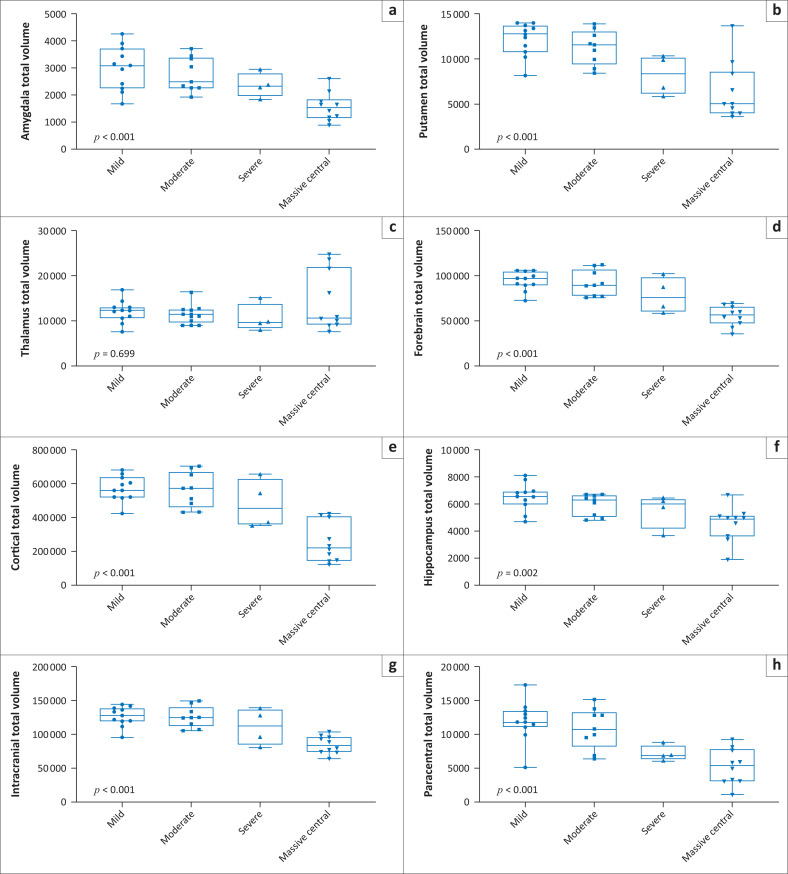
Compendium of box plot graphs for substrate injuries in patients who suffered one of the four subgroups of the Rolandic Basal Ganglia-Thalamus subtype of hypoxic-ischaemic brain injury.

We found that the average intracranial volume was reduced to the 1st percentile in all patient groups (*p* < 0.001), across all four subtypes of HIBI (Table 1, Figure 4). Most marked was the reduction in the volume of the cortical grey matter (364.2 cm^3^ – 3rd percentile, *p* < 0.001), PCL (6.5 cm^3^ – 1st percentile, *p* = 0.001) and occipital lobe (32.2 cm^3^ – 1st percentile, *p* = 0.001) volumes.

Neuroquantification of the thalamus demonstrated significant volume reduction, particularly in the RBGT subtype (1st percentile ranking). There was less significant volume reduction of the thalamic volume in the watershed (77th percentile) subtype and the mixed subtype (66th percentile). Further quantification within the RBGT subtype revealed a reduction of the thalamus volume in all RBGT or central subgroups (Figure 4).

Similarly, incremental substrate destruction could be clearly defined using measured volumes and percentile rankings for each region of interest, notably in the basal ganglia, particularly the putamina. The basal ganglia demonstrated markedly reduced overall volume (27th percentile) for patients in the RBGT subtype (Figure 4). This was more significantly reduced compared to the watershed subtype (88th percentile), where the putamen is usually spared. In the mixed injury patterns, the basal ganglia are moderately reduced (39th percentile) as there is usually some but generally less severe basal ganglia and thalamic involvement.

**FIGURE 5:**
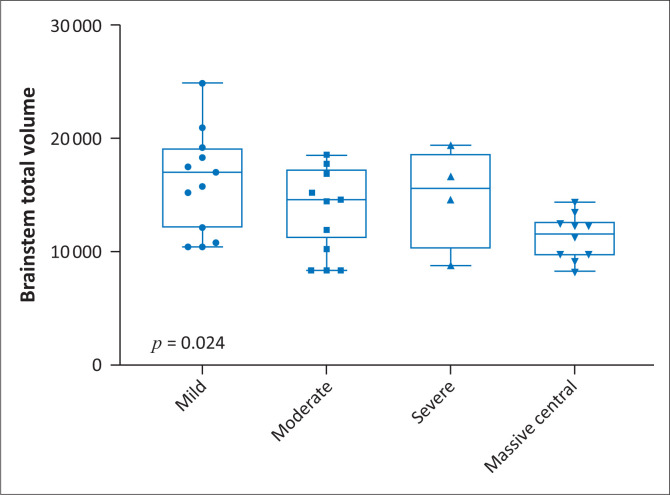
Distribution of brainstem total volume by Rolandic Basal Ganglia-Thalamus subgroups of hypoxic-ischaemic brain injury type 1.

On average, total brainstem volume was 5.30 cm^3^ greater in the mild RBGT subgroup than in the MPI subgroup (β = 5.30; *p* = 0.002, Figure 5). The brainstem volume was also reduced in the mixed pattern group (31st percentile), largely due to the RBGT component of combined injury. Notably, the watershed pattern group demonstrated almost no reduction in brainstem volume (99th percentile). This is a critical differentiating factor between the two principal patterns (Table 2 and Table 3).

**TABLE 2:** Volumetric distribution by hypoxic-ischaemic brain injury subtype 1 (Rolandic Basal Ganglia-Thalamus pattern).

Variable	Mild (*n* = 11)		Moderate (*n* = 9)		Severe (*n* = 4)		Massive paramedian (*n* = 10)		Total (*n* = 34)		*p*
	Median	IQR	Median	IQR	Median	IQR	Median	IQR	Median	IQR	
Age	7	5–9	5	4–6	7	4–11	6	3–7	5	4–7	0.358
Parasagittal Score	2	2–3	3	2–3	5	4–6	6	5–7	6	3–11	< 0.001
Amygdala Vol	3	2–4	2	2–3	2	2–3	2	1–2	2	2–3	0.001
Amygdala %	3	1–17	2	1–3	2	1–4	1	1–2	6	1–33	0.504
Basal Ganglia Vol	23	21–25	23	20–26	15	12–19	14	10–21	21	15–24	0.007
Basal Ganglia %	55	3–92	47	2–86	1	1–42	26	1–89	58	1–99	0.456
Brainstem Vol	17	12–19	15	12–17	16	12–18	12	10–12	14	11–16	0.024
Brainstem %	12	5–75	12	1–41	56	12–89	66	9–93	58	7–99	0.347
Caudate Vol	8	6–9	9	7–11	5	3–6	6	4–13	7	5–9	0.114
Caudate %	89	67–99	99	84–99	30	1–75	72	3–99	99	67–99	0.094
Forebrain Vol	967	888–1046	894	778–1028	760	618–943	566	469–652	658	525–899	< 0.001
Forebrain %	64	30–90	27	6–60	8	1–18	1	1–1	1	1–31	< 0.001
Frontal Vol	5	4–5	4	3–4	4	3–5	1	1–2	2	1–4	< 0.001
Cortical Vol	561	519–638	573	484–651	456	361–601	223	147–402	364	213–545	< 0.001
Cortical %	92	56–95	79	7–97	49	21–73	1	1–5	3	1–79	0.014
Hippocampus Vol	7	6–7	6	5–7	6	5–6	5	4–5	6	5–7	0.007
Hippocampus %	3	1–20	1	1–3	22	1–71	12	1–57	8	1–84	0.414
Intracranial Vol	1274	1186–1379	1243	1148–1333	1119	882–1332	835	738–951	962	838–1229	< 0.001
Intracranial %	1	1–15	3	1–12	5	1–10	1	1–1	1	1–1	0.056
Paracentral Vol	12	11–13	11	10–13	7	6–8	5	3–8	7	4–11	< 0.001
Paracentral %	73	21–93	67	6–88	2	1–3	1	1–3	1	1–64	0.004
Parietal Vol	141	133–156	141	116–165	113	99–145	57	37–109	93	53–136	0.001
Parietal %	93	36–98	89	44–96	64	39–90	1	1–60	14	1–91	0.124
Primary Motor Vol	35	32–38	37	34–45	29	26–36	19	11–26	23	15–33	< 0.001
Primary Motor %	97	66–99	99	97–99	99	86–99	23	1–76	65	1–98	0.039
Primary Sensory Vol	29	27–31	33	30–37	26	24–31	13	8–26	21	12–29	0.004
Primary Sensory %	89	84–98	99	96–99	99	93–99	6	1–99	82	1–99	0.095
Putamen Vol	13	11–14	12	10–13	8	6–10	5	4–8	10	6–12	0.001
Putamen %	43	1–75	1	1–34	1	1–41	1	1–1	1	1–68	0.257
Occipital Vol	59	50–60	54	42–68	43	29–58	27	16–37	32	23–58	0.005
Occipital %	22	5–73	34	1–51	6	1–11	1	1–4	1	1–26	0.116
Thalamus Vol	12	11–13	11	10–12	10	9–12	11	9–21	12	10–15	0.699
Thalamus %	1	1–11	1	1–7	2	1–4	24	2–99	32	1–99	0.056
Ventricle Vol	25	13–41	35	21–72	37	30–48	52	43–64	35	21–59	0.127
Ventricle %	95	68–99	99	82–99	99	99–99	99	99–99	99	98–99	0.011

Note: % = Percentile ranking; vol = volume measured in cm^3^.

IQR, interquartile range.

**TABLE 3:** Volumetric distribution by hypoxic-ischaemic brain injury subtype 2 (watershed pattern).

Variable	Other Combined Subgroups (*n* = 13)		Subgroup 7 (*n* = 22)		Total (*n* = 35)		*p*
	Median	IQR	Median	IQR	Median	IQR	
Patient Age (years)	5	4–10	5	4–8	5	4–7	0.629
Parasagittal Score	6	6–8	12	10–13	6	3–11	< 0.001
Amygdala Vol	3	2–3	3	2–3	2	2–3	0.609
Amygdala %	26	17–51	53	6–87	6	1–33	0.493
Basal Vol	19	13–21	20	15–27	21	15–24	0.517
Basal %	86	1–99	96	17–99	58	1–99	0.246
Brainstem Vol	14	12–16	14	13–16	14	11–16	0.838
Brainstem %	82	17–99	99	76–99	58	7–99	0.095
Caudate Vol	6	5–8	8	5–11	7	5–9	0.094
Caudate %	99	95–99	99	99–99	99	67–99	0.189
Forebrain Vol	669	576–731	534	456–634	658	525–899	0.031
Forebrain %	7	1–27	1	1–1	1	1–31	0.003
Frontal Vol	3	2–3	2	1–2	2	1–4	0.041
Frontal %	3	1–25	1	1–4	4	1–37	0.095
Cortical Vol	339	252–417	208	173–338	364	213–545	0.009
Cortical %	5	1–25	1	1–1	3	1–79	0.019
Hippocampus Vol	6	5–7	6	4–7	6	5–7	0.946
Hippocampus %	67	17–91	89	3–99	8	1–84	0.487
Intracranial Vol	938	859–1016	879	769–969	962	838–1229	0.109
Intracranial %	1	1–1	1	1–1	1	1–1	1.000
Paracentral Vol	7	4–8	4	3–6	7	4–11	0.022
Paracentral %	1	1–9	1	1–1	1	1–64	0.386
Parietal Vol	92	53–112	55	44–80	93	53–136	0.037
Parietal %	18	1–74	1	1–1	14	1–91	0.007
Primary Motor Vol	23	13–26	15	11–20	23	15–33	0.020
Primary Motor %	74	1–84	1	1–20	65	1–98	0.027
Primary Sensory Vol	22	15–25	13	11–19	21	12–29	0.037
Primary Sensory %	96	26–99	3	1–47	82	1–99	0.023
Putamen Vol	9	5–11	10	6–12	10	6–12	0.891
Putamen %	18	1–95	54	1–87	1	1–68	0.873
Occipital Vol	30	24–44	28	18–31	32	23–58	0.413
Occipital %	1	1–7	1	1–1	1	1–26	0.213
Thalamus Vol	12	9–13	12	10–14	12	10–15	0.682
Thalamus %	28	1–99	92	32–99	32	1–99	0.115
Ventricle Vol	29	18–46	36	25–67	35	21–59	0.162
Ventricle %	99	96–99	99	99–99	99	98–99	0.082

Note: % = Percentile ranking; vol = volume measured in cm^3^.

IQR, interquartile range.

**FIGURE 6:**
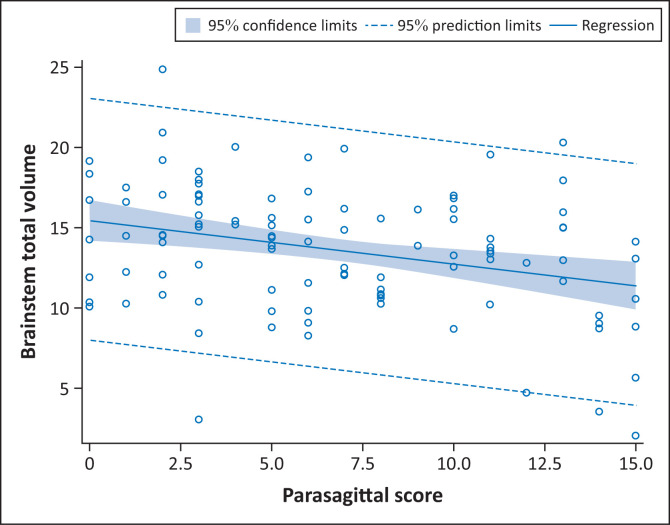
Relationship between parasagittal score and brainstem total volume (*p* < 0.001).

There were significant differences in total brainstem volume between the four groups, with the mild subgroup having a higher volume (Figure 5). In addition, we demonstrated a comparative decrease in brainstem volume with increase in parasagittal score. For every unit increase in the parasagittal score, the brainstem total volume significantly decreased by 0.27 units (β = −0.27; *p* = 0.001), (Figure 6).

On further analysis of the RBGT subtype, we found that the parasagittal score significantly increased as the degree of severity of the RBGT pattern increased (*p* < 0.001) (Table 2). A marked difference in the volumes of the most severe central subgroup, called the MPI pattern, was noted, showing a reduction in volume in most of the substrates relative to the other RBGT subgroups (Table 2). As the parasagittal score increased, we noted a correlative decrease in the brainstem volume (Figure 6). This was largely isolated to the RBGT subtype.

A note is made of the inverse relationship between the PCL volumes (12 cm^3^, 11 cm^3^, 7 cm^3^ and 5 cm^3^, *p* < 0.001) and the progressive increase in the degree of RBGT severity (73rd, 67th, 1.5th and 1st percentiles) (Table 2). For the MPI subgroup, note the correlative most significant decrease in intracranial volume at 835 cm^3^ (*p* < 0.001) and proportional enlargement of the ventricles measured at 52 cm^3^ (Table 2).

Cerebral cortical volumes showed a progressive decrease in average cortical volumes (*p* < 0.001) and respective percentile ranking for mild = 561 cm^3^ (92nd percentile), moderate = 473 cm^3^ (79th percentile), severe = 456 cm^3^ (48.5th percentile) and MPI = 223 cm^3^ (1st percentile) (Table 2). This indicates the relative sparing of the cortex in the mild injury subgroup versus globally catastrophic cortical injury in the MPI subgroup (Figure 4).

**FIGURE 7:**
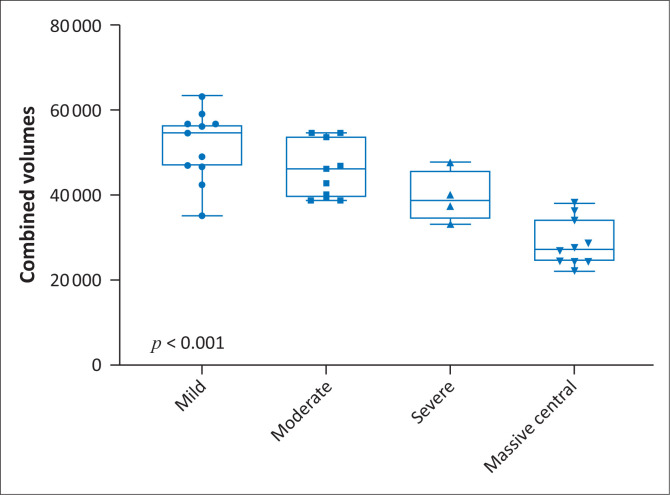
Box plot graph for combined substrate injuries (frontal pole, putamen, hippocampus, brainstem and paracentral lobule) in patients with Rolandic Basal Ganglia-Thalamus subtype of hypoxic-ischaemic brain injury.

The amygdala and hippocampal formations, which are critical components of the limbic system, were systematically reduced in average volume (*p* < 0.001) in the RBGT subtype, measuring 2 cm^3^ (1st percentile) and 6 cm^3^ (3rd percentile), respectively. This differs quite significantly from the measured average volume of the watershed subtype patients who demonstrated 2.8 cm^3^ (33rd percentile) and 6 cm^3^ (67th percentile), respectively, for the amygdala and hippocampi. Further interrogation of the amygdala injuries in the RBGT subtype showed a severe reduction in volume in all four subgroups. The percentile ranking decreased progressively from the 3rd to 2nd to 1.5th and the 1st percentile ranks for mild through to MPI subgroups (Figure 4). We subsequently combined five regions that were commonly found to be injured (the PCL, putamen, hippocampus, frontal pole and brainstem volumes). There were statistically significant differences between the four groups of the RBGT subtype in terms of the combined total volumes of the five regions described earlier; particularly, the mild subgroup had a higher volume and the MPI subgroup showed markedly reduced combined volume (Figure 7).

In the analysis of the HIBI subtype 2 (watershed pattern), the seven subgroups were individually evaluated. Subgroup 7 (where all three watershed zones were involved) was the commonest (*n* = 22). The rest of the watershed subgroups (*n* = 13) were grouped into one comparative cohort (Table 3), to more meaningfully analyse the dataset for all these patients. The parasagittal score was much higher (12 vs 6) for subgroup 7, consistent with the more extensive destruction of the parasagittal cortex and greater substrate injury in the superior convexity of the cerebrum (*p* < 0.001, Table 3). Similarly, multiple substrates showed a remarkable average reduction in volume in subgroup 7 versus all other subgroups for the following regions: forebrain volume (534 cm^3^ vs 669 cm^3^), cortical volume (208 cm^3^ vs 339 cm^3^), parietal lobe volume (55.5 cm^3^ vs 92 cm^3^), primary motor cortex volume (15 cm^3^ vs 22.5 cm^3^) and primary sensory cortex volume (13 cm^3^ vs 22 cm^3^) (Table 3).

Notably, the brainstem was not reduced in volume in this subtype of HIBI (across all seven subgroups). Even in the most severe subtype of watershed injury (subgroup 7), where all three watershed zones were involved, the brainstem volumes averaged at the 99th percentile ranking (*p* = 0.095). In this study, the watershed subtype of HIBI was, therefore, not associated with any significant brainstem volume reduction (Table 3).

## Discussion

There is increasing medicolegal litigation internationally, and the burden of HIBI is becoming even more pronounced in LMIC.^2^ The structural injuries of HIBI assessed visually by a radiologist are associated with poor neurodevelopmental outcomes.^22^ This study demonstrated a severe reduction in overall brain volume across the spectrum of HIBI subtypes when using an accredited neuroquantification software program. The average intracranial volume was reduced to the first percentile in all patient groups (*p* < 0.001). This is a key finding, quantitatively proving the significant morbidity of HIBI, irrespective of the subtype of injury sustained.

The complexity of brain injuries in HIBI demands the specification of the interrogating algorithms for this category of patients. Standardisation of imaging protocols is critical for obtaining usable datasets for volumetric analyses and accurately quantifying substrate injuries. A review of the available segmentation methods applicable to cerebral palsy concluded that atlas-based comparison in areas of substantial morphological injury may not suffice in accurately quantifying substrate destruction.^23^ In this study, the available NeuroQuant^®^ (Cortechs Labs, Inc., San Diego, CA, US) age-matched atlases allow for the categorisation of the percentile rankings of study participants. Percentile rankings are useful in describing the degree of difference between affected children and their peers. These are useful if correlated with clinical parameters.

The typical patterns of HIBI pertain to the involvement of high metabolic substrates and watershed cerebral injuries or a combination thereof. The described patterns of HIBI, though repeated in individuals and following fairly expected substrate destruction, will produce a unique phenotype dependant on individual genetic factors, inflammatory pathways, neurotransmitter cascades and neuroplasticity.^14^ Serial imaging with substrate size measurement enables quantification of the end result of perinatal HIBI.^4^ This study, quantified brain injuries, and when measured against age-matched atlases, showed how severely the HIBI patients were affected.

For the RBGT subtype, there was a progressive increment in the volumetric measured substrate destruction. A stepwise gradation of injury was shown from the mild through the moderate, severe and MPI subgroups for the following substrates: basal ganglia (putamen), lobar volumes (parietal, occipital), cerebral cortex, PCLs and thalami. In the previously described most severe subgroup of RBGT injury, referred to as MPI, the substrate damage was most marked.

Brainstem injuries in hypoxic-ischaemic encephalopathy are often underestimated as radiologists focus on the more commonly described patterns of cerebral injuries.^24^ Despite the brainstem rarely demonstrating signal changes on conventional sequences, this study demonstrated that the brainstem was severely reduced in volume in the RBGT group where, probably consequent on the long tract (especially corticospinal) involvement, there was a secondary reduction in the fibre tract volumes with a smaller calibre brainstem. In those children with the watershed subtype pattern of cerebral injury, there was relative sparing of the brainstem with almost no observable volume reduction across the spectrum of all seven subgroups. This is significantly different to what was found in the RBGT subtype, where the brainstem was reduced in volume to an average 15th percentile. This is an important differentiating feature not previously reported.

The thalamus has been defined as a very important neural hub, linking several diverse neural circuits in the human brain. Thalamic injuries in different HIBI subtypes are well described. The average thalamic volume for all RBGT patients was measured at the 1st percentile ranking (*p* < 0.001), confirming that this substrate is severely destroyed in patients sustaining this specific subtype of HIBI. Across all the RBGT subgroups, there was marked thalamic volume reduction in all patients. This confirms that injury of this substrate is a key feature of the RBGT pattern where the VPL nucleus is principally injured, and progressive necrosis of the other thalamic nuclei is seen in more severe subgroups of RBGT injury.

This study also validates, by direct substrate volume measurement, the previously purported view that the thalamus is significantly involved in the basal ganglia-thalamus pattern of injury.^25^ There is also relatively less destruction of the thalamus in patients who suffered the watershed pattern of injury. The previous description of the thalamus L-sign^21^ signifies injury of the posterolateral thalamic margin (including the thalamic reticular nucleus, lateral geniculate body and pulvinar) adjacent to the corticospinal long tract. This sign is seen in the watershed subtype or combined hypoglycaemic or partial prolonged HIBI injuries. This study predicts a less marked thalamic volume reduction in a watershed subtype of HIBI than in the RBGT subtype.

The occipital lobe, in particular, the visual cortex, has also been identified as a high metabolic zone^14^ of the brain, often associated with central subtype injuries. This is corroborated in the current study where there was a stepwise reduction in average occipital lobe volume as the central injury severity increased. There was a less significant but similar trend for the parietal lobe volumes in RBGT patients. The average volumetric measurement of the parietal and occipital lobes for patients in the MPI subgroup was at the 1st percentile ranking.

This study has shown that by combining five regions of interest (frontal pole, putamen, hippocampus, brainstem and PCL volumes) with particular reference to the RBGT subtype of HIBI, there was a strong correlation between the RBGT subtype and combined reduction in volume in these substrates. This was a unique finding and may provide insight into the timing and mechanism for HIBI. The possibility that each of these substrates may be injured in other pathological processes remains a reality; however, the combined injury of high metabolic substrates favours the RBGT subtype of HIBI. Further larger multicentre interrogation of this finding is warranted.

The corpus callosum (CC), although an important white matter structure, often found to be injured in children with HIBI, was excluded as a region of interest in this study. Prior studies evaluating CC volumetry confirmed injury of this commissural substrate in cases of profound HIBI.^26^ Measurement of corpus callosal fibre tracts is problematic due to the lack of clarity as to where one terminates the measurement at the lateral edge of the tract, that is, where does the CC actually end in its lateral aspect as it blends with the adjacent white matter. Accurate measurements of the entire CC can, therefore, not be correctly calculated. Most studies^27,28^ have shown the secondary effect of thinning parts of the CC as measured in midline sagittal slices. For the purposes of this study, and due to the lack of age-matched percentile callosal volumetric measurements, it was resolved to exclude CC volumetry.

An important limitation of the study is the number of patients recruited for the broad categories and subtypes of HIBI. In addition, the age threshold of 3 years limited the number of patients for whom substrate volumes could be quantified. Larger sample groups and lowering the age for neuroquantification by expanding comparative atlases would add to the power of substrate evaluations. This could be further enhanced by larger multicentre studies to compare HIBI subtypes. Multicystic encephalomalacia and severe mixed subtype patterns following global brain injury with spongiosis of multiple substrates render volumetric analysis technically difficult due to the lack of measurable substrates.

## Conclusion

In recent years, the use of AI applications has grown exponentially worldwide and particularly in radiology. Using neuroquantification software, we have more accurately measured brain injuries in children presumed to have sustained term neonatal HIBI. All patients who suffered HIBI demonstrated reduced brain volumes, with the average intracranial volume reduced to the first percentile in all HIBI subtypes (*p* < 0.001). Definitive substrate injury patterns are more pronounced in certain subtypes of HIBI and in the respective subgroups. A stepwise gradation with increasing substrate volume reduction was shown in the RBGT subgroups from the mild to most severely injured. Brainstem volume reduction was isolated to the RBGT subtype and unaffected in the watershed subtype patterns of HIBI. Similarly, thalamic, amygdala and occipital lobe volume reduction was a feature of the RBGT subtype with relatively normal volumes in watershed subtypes. Combined injury with reduced volumes of five regions of interest (frontal pole, putamen, hippocampus, brainstem and PCL) was repeatedly noted in patients with RBGT subtype of HIBI.

The AI algorithms used in this study may, in the correct clinical setting, be extrapolated to support stratification of HIBI.

## References

[1] Volpe JJ. Neurology of the newborn. 6th ed. Philadelphia, PA: Saunders; 2018, Chapter 18 and 19.

[2] Nanyunja C, Sadoo S, Mambule I, et al. Protocol for the Birth Asphyxia in African Newborns (Baby BRAiN) Study: A Neonatal Encephalopathy Feasibility Cohort Study [version 1; peer review: 2 approved]. Gates Open Res. 2022;6:10. 10.12688/gatesopenres.13557.1 35614965 PMC9110736

[3] Barkovich AJ, Truwit CL. Brain damage from perinatal asphyxia: Correlation of MR findings with gestational age. AJNR Am J Neuroradiol. 1990;11:1087–1096. 2124034 PMC8332119

[4] Rutherford M, Malamateniou C, McGuinness A, Allsop J, Biarge MM, Counsell S. Magnetic resonance imaging in hypoxic-ischaemic encephalopathy. Early Hum Dev. 2010;86(6):351–360. 10.1016/j.earlhumdev.2010.05.014 20541877

[5] De Vries LS, Groenendaal F. Patterns of neonatal hypoxic–ischaemic brain injury. Neuroradiology. 2010;52(6):555–566. 10.1007/s00234-010-0674-9 20390260 PMC2872019

[6] Miller SP, Ramaswamy V, Michelson D, et al. Patterns of brain injury in term neonatal encephalopathy. J Pediatr. 2005;146(4):453–460. 10.1016/j.jpeds.2004.12.026 15812446

[7] Sie L, Van der Knaap M, Oosting J, De Vries L, Lafeber H, Valk J. MR patterns of hypoxic-ischemic brain damage after prenatal, perinatal or postnatal asphyxia. Neuropediatrics. 2000;31(03):128–136. 10.1055/s-2000-7496 10963099

[8] Ranck JB, Windle WF. Brain damage in the monkey, macaca mulatta, by asphyxia neonatorum. Exp Neurol 1959;1(2):131–150. 10.1016/0014-4886(59)90032-9 13663899

[9] Mota-Rojas D, Villanueva-García D, Solimano A, Muns R, Ibarra-Ríos D, Mota-Reyes A. Pathophysiology of perinatal asphyxia in humans and animal models. Biomedicines. 2022;10(2):347. 10.3390/biomedicines10020347 35203556 PMC8961792

[10] Windle WF. Brain damage at birth. JAMA. 1968;206;1967. 10.1001/jama.206.9.1967 4972328

[11] Myers RE. Two patterns of perinatal brain damage and their conditions of occurrence. Am J Obstet Gynecol. 1972;112(2):246–276. 10.1016/0002-9378(72)90124-X 4621486

[12] Gunn AJ, Bennet L. Fetal hypoxia insults and patterns of brain injury: insights from animal models. Clin Perinatol. 2009;36(3):579–593. 10.1016/j.clp.2009.06.007 19732615 PMC2767254

[13] Lai MC, Yang SN. Perinatal hypoxic-ischemic encephalopathy. J Biomed Biotechnol 2011;2011:609813. 10.1155/2011/609813 21197402 PMC3010686

[14] Misser SK, Barkovich AJ, Lotz JW, Archary M. A pictorial review of the pathophysiology and classification of the magnetic resonance imaging patterns of perinatal term hypoxic ischemic brain injury – What the radiologist needs to know… S Afr J Radiol. 2020;24(1):1915. 10.4102/sajr.v24i1.1915 PMC7670012 33240541

[15] Pringle C, Kilday JP, Kamaly-Asl I, Stivaros SM. The role of artificial intelligence in paediatric neuroradiology. Pediatr Radiol. 2022;52(11):2159–2172. 10.1007/s00247-022-05322-w 35347371 PMC9537195

[16] Iwasaki N, Hamano K, Okada Y, et al. Volumetric quantification of brain development using MRI. Neuroradiology. 1997;39(12):841–846. 10.1007/s002340050517 9457706

[17] Reid LB, Pagnozzi AM, Fiori S, Boyd RN, Dowson N, Rose SE. Measuring neuroplasticity associated with cerebral palsy rehabilitation: An MRI based power analysis. Int J Dev Neurosci. 2017;58:17–25. 10.1016/j.ijdevneu.2017.01.010 28130065

[18] Faria AV, Hoon A, Stashinko E, et al. Quantitative analysis of brain pathology based on MRI and brain atlases—Applications for cerebral palsy. NeuroImage. 2011;54(3):1854–1861. 10.1016/j.neuroimage.2010.09.061 20920589 PMC3008311

[19] Misser SK, Lotz JW, Zaharie SD, et al. A proposed magnetic resonance imaging grading system for the spectrum of central neonatal parasagittal hypoxic–ischaemic brain injury. Insights Imaging. 2022;13:11. 10.1186/s13244-021-01139-7 35072815 PMC8787015

[20] Chacko A, Andronikou S, Mian A, et al. Cortical ischaemic patterns in term partial-prolonged hypoxic-ischaemic injury – The inter-arterial watershed demonstrated through atrophy, ulegyria and signal change on delayed MRI scans in children with cerebral palsy. Insights Imaging. 2020;11(1):53. 10.1186/s13244-020-00857-8 32232679 PMC7105592

[21] Misser SK, Lotz JW, Van Toorn R, Mchunu N, Archary M, Barkovich AJ. Thalamus L-sign: A potential biomarker of neonatal partial, prolonged hypoxic-ischemic brain injury or hypoglycemic encephalopathy? AJNR Am J Neuroradiol. 2022;43(6):919–925. 10.3174/ajnr.A7511 35589136 PMC9172948

[22] De Vries LS, Jongmans MJ. Long-term outcome after neonatal hypoxic-ischaemic encephalopathy. Arch Dis Child Fetal Neonatal Ed. 2010;95(3):F220–F224. 10.1136/adc.2008.148205 20444814

[23] Pagnozzi AM, Gal Y, Boyd RN, et al. The need for improved brain lesion segmentation techniques for children with cerebral palsy: A review. Int J Dev Neurosci. 2015;47(Part B):229–246. 10.1016/j.ijdevneu.2015.08.004 26394278

[24] Quattrocchi CC, Errante Y, Rossi Espagnet MC, et al. Magnetic resonance imaging differential diagnosis of brainstem lesions in children. World J Radiol. 2016;8(1):1–20. 10.4329/wjr.v8.i1.1 26834941 PMC4731345

[25] Elsingergy MM, Worede F, Venkatakrishna S, Andronikou S. Deep nuclei injury distribution in isolated “basal ganglia-thalamus” (BGT) versus combined “BGT and watershed” patterns of hypoxic-ischaemic injury (HII) in children with cerebral palsy. Clin Radiol. 2022;77(11):825–832. 10.1016/j.crad.2022.04.019 35649736

[26] Stivaros SM, Radon MR, Mileva R, et al. Quantification of structural changes in the corpus callosum in children with profound hypoxic-ischaemic brain injury. Pediatr Radiol. 2016;46(1):73–81. 10.1007/s00247-015-3444-3 26403618 PMC4706576

[27] Andronikou S, Pillay T, Gabuza L, et al. Corpus callosum thickness in children: An MR pattern-recognition approach on the midsagittal image. Pediatr Radiol. 2015;45(2):258–272. 10.1007/s00247-014-2998-9 25173405

[28] Garhwal A, Patil A. Role of magnetic resonance imaging in biometric evaluation of corpus callosum in hypoxic ischemic encephalopathy patients. J Sci Soc. 2017;44(2):67–75. 10.4103/jss.JSS_30_16

